# The role of the lactate dehydrogenase-to-albumin ratio in predicting renal prognosis in Chinese IgA nephropathy patients: a retrospective cohort study

**DOI:** 10.3389/fendo.2026.1592948

**Published:** 2026-03-26

**Authors:** Siqing Wang, Lingqiu Dong, Huan Zhou, Wei Qin

**Affiliations:** Division of Nephrology, Department of Medicine, West China Hospital of Sichuan University, Chengdu, Sichuan, China

**Keywords:** IgA nephropathy, lactate dehydrogenase-to-albumin ratio, pathological lesions, renal survival, renal prognosis

## Abstract

**Background:**

Immunoglobulin A nephropathy (IgAN) is one of the most common types of primary glomerulonephritis and is an important cause of end-stage renal disease (ESRD) worldwide. Inflammation has been shown to be associated with its basic pathogenesis. The lactate dehydrogenase-to-albumin ratio (LAR), a novel marker of inflammation and nutritional status, has been studied in various diseases. However, whether the LAR also plays a critical role in Chinese patients with IgAN remains unknown. Thus, we conducted this retrospective study to evaluate the role of the LAR in predicting clinicopathologic changes and disease prognosis in IgAN patients.

**Methods:**

A total of 1,276 patients with biopsy-proven IgAN were enrolled in this study. The patients were grouped into a high LAR group (LAR ≥4.05, *n* = 738) and a low LAR group (LAR <4.05, *n* = 538) based on the cutoff value of the LAR with regard to the Youden index. The study endpoint was a composite endpoint that referred to ESRD and/or an estimated glomerular filtration rate (eGFR) that decreased by more than 50% compared with baseline. The predictive value was determined by the area under the receiver operating characteristic curve (AUROC). Kaplan–Meier and Cox proportional hazards analyses were performed to evaluate the value of the LAR in predicting renal progression and patient prognosis.

**Results:**

IgAN patients with a high LAR had an increased incidence of anemia and increased levels of proteinuria, serum creatinine, and serum lipids. Multivariate Cox regression analysis indicated that a high LAR was an independent risk factor for IgAN even after adjustment for important clinicopathological parameters (HR = 1.844, 95% CI = 1.138–2.988, *p* = 0.013). Kaplan–Meier analysis revealed that a high LAR was significantly associated with a poor renal prognosis in patients with IgAN (*p* < 0.001). According to subgroup analysis stratified by sex, renal function, treatment, anemia status, or proteinuria level, a high LAR was consistently related to a worse renal outcome.

**Conclusion:**

An elevated LAR affects renal progression and prognosis in patients with IgAN and could be a novel marker for the management of IgAN patients in the future.

## Introduction

Immunoglobulin A nephropathy (IgAN) is one of the most common types of primary glomerulonephritis worldwide, and it includes a range of clinical presentations, from asymptomatic microscopic hematuria or proteinuria to rapid kidney failure (1). Furthermore, it is associated with considerable socioeconomic resources, especially in Asia and the Western world (2). Thus, it is worthwhile to explore new prognostic factors that evaluate the high risk of a progressive reduction in renal function in IgAN patients to improve disease management.

Lactate dehydrogenase (LDH), which converts pyruvate to lactate, is a common enzyme in many types of cells and contributes to energy metabolism (3). Moreover, the response of the serum albumin (ALB) concentration to systemic inflammation and nutritional status has been explored (4). In addition, chronic inflammation reportedly participates in the pathogenesis of IgAN (5). The lactate dehydrogenase-to-albumin ratio (LAR) has been evaluated as a prognostic factor for several diseases. The LAR could be used as an independent prognostic factor for in-hospital mortality in lower respiratory tract infection patients in the emergency department (6). The preoperative LAR could predict overall survival and disease-recurrence-free survival in patients with urothelial carcinoma of the bladder after radical cystectomy (7). The greater the LAR is, the greater the 28-day, 90-day, and in-hospital death rates are for sepsis-associated acute kidney injury patients in the intensive care unit (8).

However, no studies have been conducted on whether the combination of LDH and ALB, which reflects both inflammation and nutritional status, is a prognostic factor for composite renal outcome in IgAN patients.

Thus, we performed the present retrospective study on 1,276 patients with IgAN to provide more evidence about the relationships between the LAR and renal function, pathological lesions, disease progression, and prognosis.

## Materials and methods

### Patients

**Figure 1:**
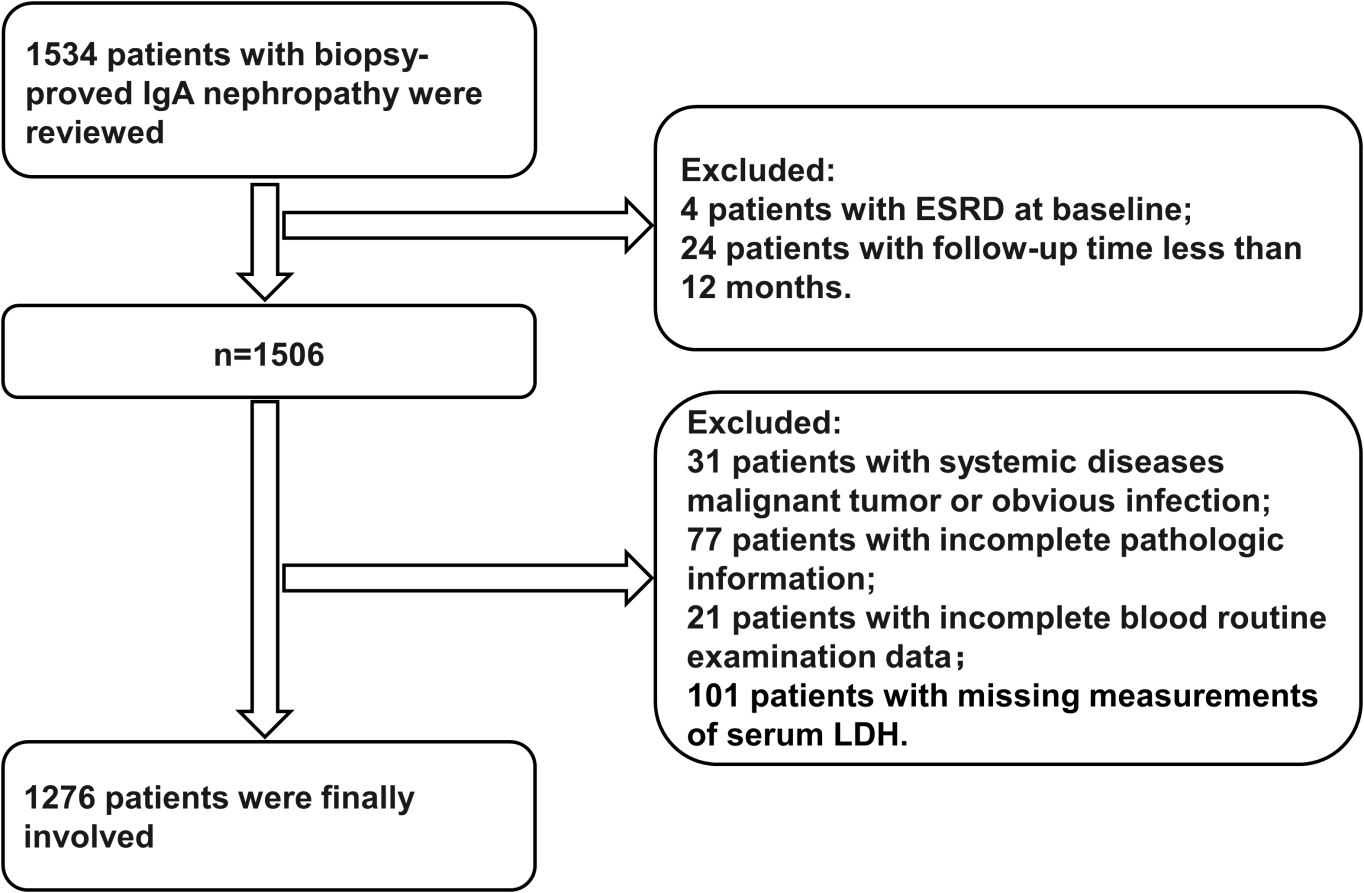
Flowchart of patients included in this study. ESRD, end-stage renal disease.

This study reviewed 1,276 patients with renal-biopsy-proven IgAN at West China Hospital of Sichuan University from March 2009 to December 2018. According to the pathological results of the renal biopsy, which shows the predominance of IgA deposits in the glomerular mesangium, either alone or with IgG, IgM, or complement C3, we made the diagnosis of IgAN (9). Patients with the following characteristics were excluded before a series of analyses: (1) patients with systemic diseases (systemic lupus erythematosus, diabetes mellitus, Henoch–Schönlein purpura, and liver dysfunction or cirrhosis), malignant tumors, or obvious infection (31 patients), (2) patients with incomplete pathologic information (77 patients), (3) patients with missing routine blood examination data (21 patients) and patients lacking measurements of serum LDH (101 patients), (4) patients with ESRD during the period of renal biopsy (four patients), and (5) patients with a follow-up time of less than 12 months before reaching the composite renal outcome (24 patients) (**Figure 1**).

This retrospective study was performed according to the Helsinki Declaration and was authorized by the Ethics Committee of West China Hospital of Sichuan University (2019-33). Informed consent was waived due to the retrospective nature of the study.

### Clinical data

Demographic data (gender, age) as well as other important clinical information (such as hypertension history) were obtained from the hospital electronic information system. Initial blood and urine laboratory results [serum creatinine, hemoglobin, lymphocyte count, serum albumin, serum lipid, uric acid, proteinuria, and estimated glomerular filtration rate (eGFR)] prior to renal biopsy were also collected. The mean arterial pressure (MAP) was defined as the diastolic pressure plus one-third of the pulse pressure (10). The eGFR was calculated using the CKD-EPI equation (11). The LAR was computed as the initial serum LDH (U/L)/serum albumin (g/L) ratio. Anemia was defined as a hemoglobin concentration of less than 120 g/L in men and less than 110 g/L in women (12).

### Pathological data

Renal biopsy samples were collected and evaluated by experienced pathologists and nephrologists according to the Oxford classification of IgAN based on the results of light microscopy (hematoxylin–eosin, periodic acid–Schiff, Masson, and periodic acid–Schiff/methenamine staining), immunofluorescence (IgA, IgG, IgM, C3, C4, and C1q), and electron microscopy. The Oxford classification of IgAN includes mesangial hypercellularity (M0/M1), endocapillary hypercellularity (E0/E1), segmental glomerulosclerosis (S0/S1), tubular atrophy/interstitial fibrosis (T0/T1/T2), and cellular or fibrocellular crescents (C0/C1/C2) (13).

### Treatment data and endpoints

According to the treatment of all patients after renal biopsy, they were divided into a supportive treatment group and a prednisone or other immunosuppressive agent group. The study endpoint was the composite endpoint, which indicates end-stage renal disease (ESRD: eGFR less than 15 mL/min/1.73 m^2^ or undergoing renal replacement treatment) and/or an eGFR decrease of more than 50% compared with baseline.

### Evaluation of the predictive value of the LAR

The discriminatory power of the predictive value for the composite renal outcome was tested by the area under the receiver operating characteristic curve (AUROC), and the optimal cutoff point of the LAR was obtained by calculating the Youden index (14), which was calculated as “sensitivity + specificity – 1”. We selected the optimal cutoff value based on the maximum Youden index.

### Statistical analysis

All statistical analyses were performed using IBM SPSS software, version 26.0 (IBM Corp., Armonk, NY, USA), and R version 4.2.1 (http://www.Rproject.org). Categorical variables are described as counts and percentages and were analyzed by *χ^2^* test or Fisher’s exact test. Continuous variables are presented as the mean ± standard or the median with interquartile ranges (25th and 75th percentiles) and were analyzed using Student’s *t*-test or the nonparametric test. The relationships between the LAR and other important clinicopathologic parameters were explored by correlation tests. In addition, the area under the receiver operating curve (AUC) was also calculated to assess the discrimination ability of the LAR. The ability of the LAR at different times to predict the composite renal risk was evaluated with a time-dependent receiver operating characteristic (td-ROC) curve. In addition, renal survival among the different groups was compared via the Kaplan–Meier method. Cox regression analysis was conducted to discriminate the risk factors for the composite renal outcome. The results are expressed as hazard ratios (HRs) and 95% confidence intervals (CIs); *p <*0.05 was considered to indicate statistical significance.

## Results

### Predictive value of the LAR for renal risk

**Figure 2:**
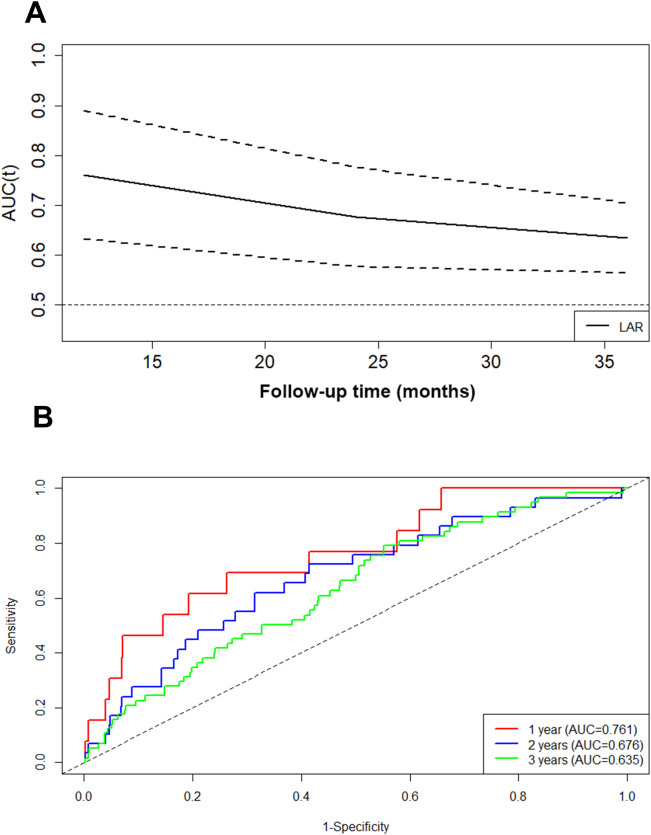
Ability of the LAR at different times to predict the composite renal outcome evaluated with a time-dependent receiver operating characteristic curve **(A, B)**.

The AUROC of the LAR was 0.646 for all patients. In addition, the area under the curve (td-AUC) of the time-dependent receiver operating characteristic curve of the LAR for predicting renal risk was 0.761 at 1 year, 0.676 at 2 years, and 0.635 at 3 years (**Figures 2A, B**). Moreover, ROC analysis revealed that the optimal cutoff LAR for predicting renal risk in patients with IgAN was 4.05.

### Demographic and clinicopathological characteristics

As outlined in **Table 1**, the mean age of all patients at the time of renal biopsy was 33 years, and 44.9% were men. The proportion of hypertension in all patients was 28.4%. With respect to renal function, the median eGFR and proteinuria were 92.07 mL/min/1.73 m^2^ and 1.41 g/day, respectively. The median LAR was 4.24 in all patients. Thus, the patients were divided into a high LAR group (LAR ≥ 4.05, *n* = 738) and a low LAR group (LAR < 4.05, *n* = 538) according to the optimal cutoff value of the ROC analysis. Compared with those in the low LAR group, patients in the high LAR group had an increased rate of anemia (11.2% *vs*. 16.4%) and increased use of prednisone or other immunosuppressive agents (44.2% *vs*. 70.7%). In addition, patients with a greater LAR were more likely to have higher levels of proteinuria, serum creatinine, total cholesterol, and triglycerides. With respect to pathologic lesions, we found that patients with greater LAR had greater tubular atrophy/interstitial fibrosis and more cellular or fibrocellular crescents.

**Table 1:** Demographic and clinicopathological characteristics of 1276 IgAN patients.

Parameters	All patients N=1276	Group1 (LAR<4.05) n=538	Group2 (LAR≥4.05) n=738	P value
Male (%)	573 (44.9%)	257 (47.8%)	316 (42.8%)	0.079
Age (years)	33 (26,42)	31 (25,40)	35 (26,44)	<0.001
MAP (mmHg)	96.33 (88.67,105.67)	95.67 (87.33,104.33)	96.67 (89.67,107.33)	0.002
Hypertension (%)	363 (28.4%)	128 (23.8%)	235 (31.8%)	0.002
Anemia (%)	181 (14.2%)	60 (11.2%)	121 (16.4%)	0.008
Proteinuria (g/d)	1.41 (0.73,2.99)	1 (0.5,1.71)	2 (1,3.58)	<0.001
24h-proteinuria≥1g/d	860 (67.4%)	277 (51.5%)	538 (79%)	<0.001
URBC (/HP)	19 (7,59.75)	18 (6,53)	19 (7,64)	0.096
ALB (g/L)	40.05 (36.1,43.2)	42.6 (39.8,45)	37.55 (33.1,41.33)	<0.001
LDH (IU/L)	171 (149,198)	148 (134,159)	191.5 (173,219)	<0.001
LAR	4.24 (3.63,5.3)	3.54 (3.2,3.77)	5.04 (4.47,6.32)	<0.001
SCr (umol/L)	84 (65.93,110)	79.25(63.23,99.65)	87.15(67.23, 119.85)	<0.001
eGFR (mL/min/1.73 m^2^)	92.07 (65.78,116.15)	100.17 (78.49,119.91)	83.08 (56.27,111.81)	<0.001
CKD stages				<0.001
Stage 1	667 (52.3%)	332 (61.7%)	335 (45.4%)	
Stage 2	338 (26.5%)	141 (26.2%)	197 (26.7%)	
Stage 3	222 (17.4%)	59 (11%)	163 (22.1%)	
Stage 4	49 (3.8%)	6 (1.1%)	43 (5.8%)	
UA (umol/L)	364 (301,440.3)	350 (289,422)	381 (306,453)	<0.001
Serum triglyceride	1.52 (1.06,2.21)	1.33(0.96,1.99)	1.65 (1.15,2.34)	<0.001
Serum total cholesterol	4.77 (4.06,5.66)	4.43 (3.8,5.07)	5.17 (4.33,6.1)	<0.001
Pathological lesions				
M				<0.001
0	300 (23.5%)	153 (28.4%)	147 (19.9%)	
1	976 (76.5%)	385 (71.6%)	591 (80.1%)	
E				<0.001
0	1217 (95.4%)	527 (98%)	690 (93.5%)	
1	59 (4.6%)	11 (2%)	48 (6.5%)	
S				0.280
0	495 (38.8%)	218 (40.5%)	277 (37.5%)	
1	781 (61.2%)	320 (59.5%)	461 (62.5%)	
T				<0.001
0	1013 (79.4%)	463 (86.1%)	550 (74.5%)	
1	218 (17.1%)	65 (12.1%)	153 (20.7%)	
2	45 (3.5%)	10 (1.8%)	35 (4.7%)	
C				<0.001
0	998 (78.2%)	450 (83.6%)	548 (74.2%)	
1	234 (18.3%)	80 (14.9%)	154 (20.9%)	
2	44 (3.4%)	8 (1.5%)	36 (4.9%)	
Treatment				<0.001
Support treatment (%)	516 (40.4%)	300 (55.8%)	216 (29.3%)	
Prednisone or other immunosuppressive agents (%)	760 (59.6%)	238 (44.2%)	522 (70.7%)	

MAP, mean arterial pressure; URBC, urinary red blood cell counts; ALB, albumin; LDH, Lactate dehydrogenase; LAR, lactate dehydrogenase to albumin ratio; SCr, serum creatinine; eGFR, estimated glomerular filtration rate; CKD, chronic kidney disease; UA, uric acid; M, mesangial proliferation; E, endocapillary proliferation; S, segmental sclerosis; T, tubular atrophy/interstitial fibrosis; C, crescents.

### Association of the LAR with clinical parameters

Correlation analyses were performed, and the results demonstrated that the LAR was positively correlated with proteinuria (*r* = 0.516, *p* < 0.001) and negatively related to the eGFR (*r* = -0.146, *p* < 0.001).

### LAR and renal prognosis in patients with IgAN

**Figure 3:**
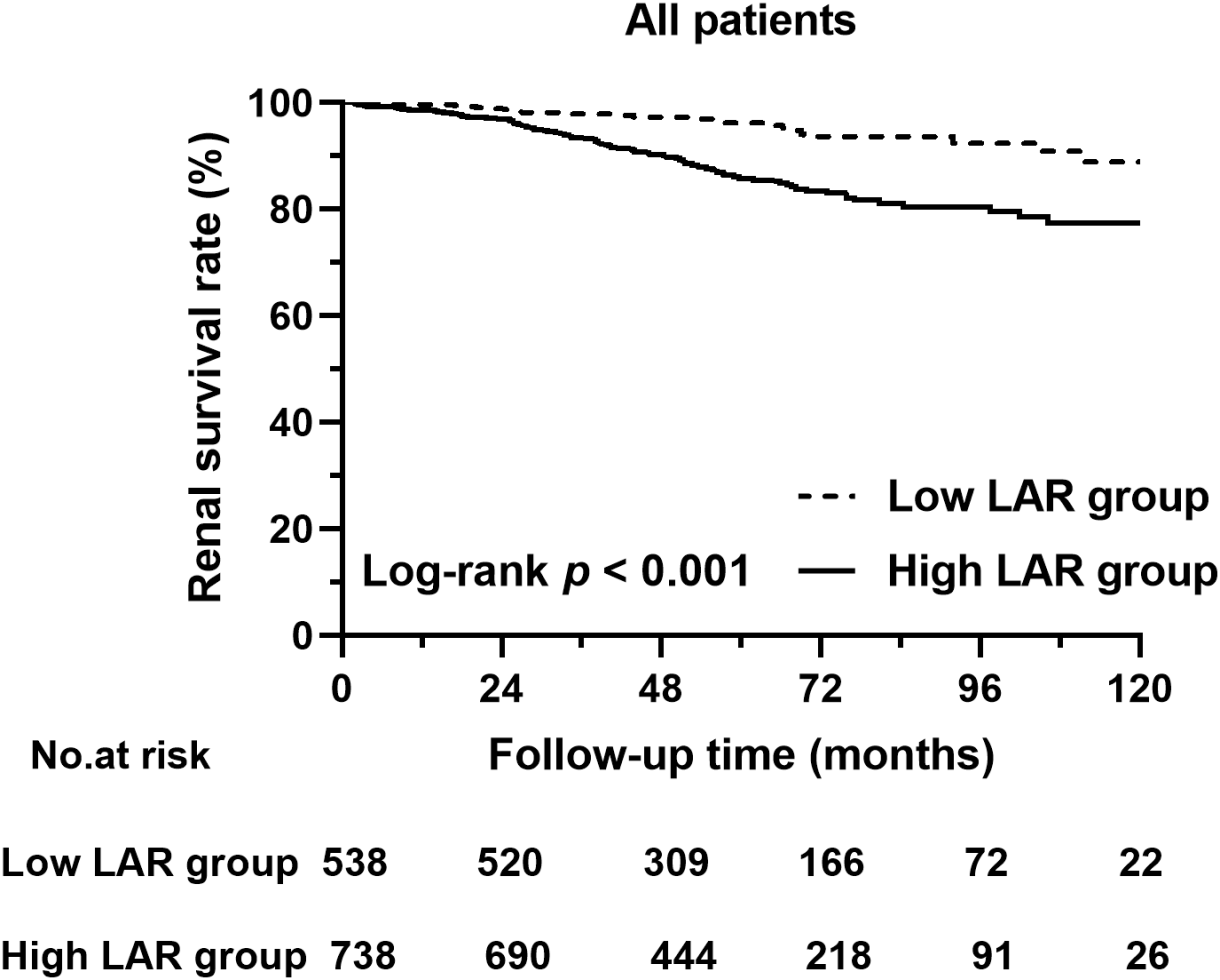
Kaplan–Meier curves of the renal survival rate in patients with different LARs.

Univariate Cox regression analysis revealed that a high LAR was related to severe composite renal risk (HR, 3.127; 95% CI, 2.004–4.878; *p* < 0.001). Moreover, multivariate Cox regression analysis suggested that a high LAR was associated with increased composite renal risk (HR, 1.844; 95% CI, 1.138–2.988; *p* = 0.013) after adjustment for age, sex, hypertension, eGFR, proteinuria greater than 1 g/day, anemia, a serum ALB concentration less than 30 g/L, treatment, and the Oxford MEST-C score (**Table 2**). The Kaplan–Meier analysis also indicated that patients with a greater baseline LAR had a significantly greater composite renal risk (*p* < 0.001, **Figure 3**).

**Table 2:** Associations of LAR with IgAN progression.

	HR (95%CI)	P value
Univariate	3.127 (2.004-4.878)	<0.001
Model 1	1.743 (1.084-2.804)	0.022
Model 2	1.723 (1.067-2.783)	0.026
Model 3	1.844 (1.138-2.988)	0.013

LAR was analyzed as a categorial variable (high LAR vs low LAR) and data are reported as hazard ratio (HR) and 95% confidence interval (CI).

Model 1, adjusted for baseline age, gender, hypertension, e-GFR, 24h-proteinuria≥1g/d, hypoalbuminemia and anemia. .

Model 2, adjusted for covariates in model 1 plus pathological lesions (MEST-C).

Model 3, adjusted for covariates in model 2 plus treatment.

**Figure 4:**
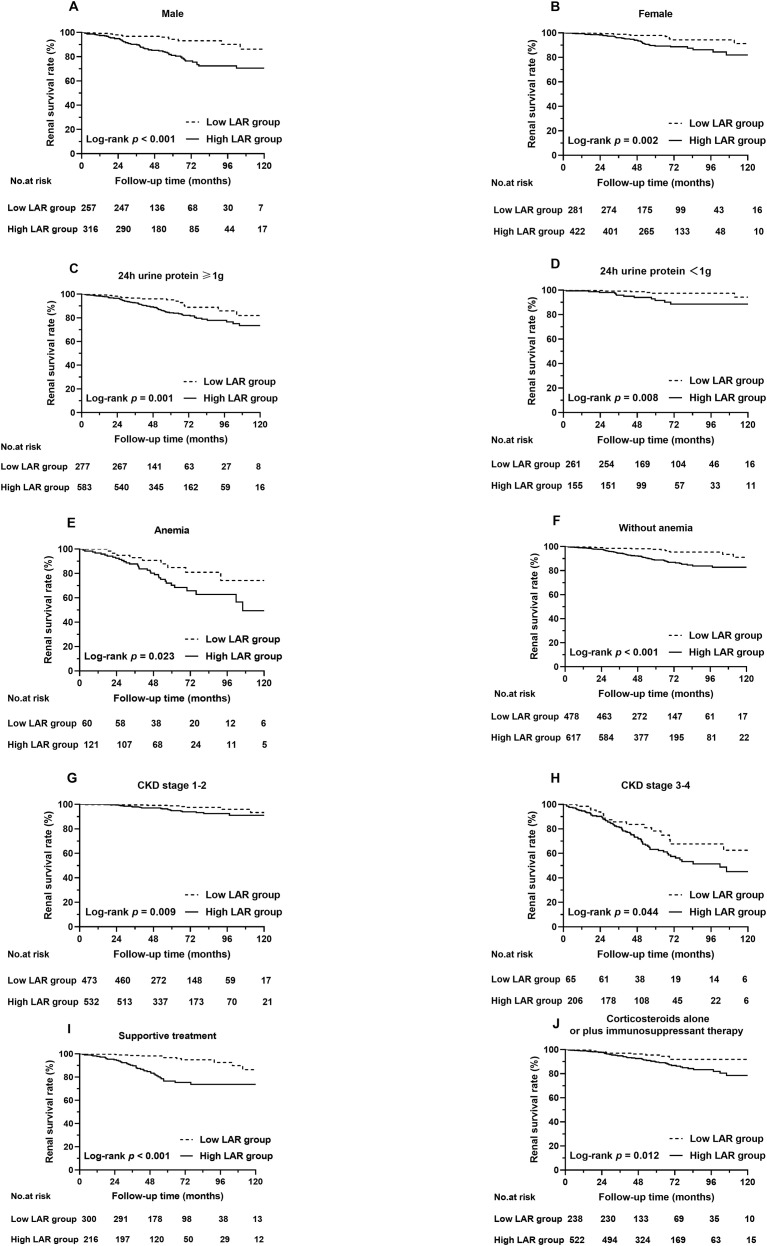
Kaplan–Meier analysis of the composite renal outcome in patients with different LARs. **(A)** Renal survival rates in male patients, **(B)** renal survival rates in female patients, **(C)** renal survival rates in patients with 24-h urine protein ≥1 g, **(D)** renal survival rates in patients with 24-h urine protein <1 g, **(E)** renal survival rates in patients with anemia, **(F)** renal survival rates in patients without anemia, **(G)** renal survival rates in patients with CKD stage 1 to 2, and **(H)** renal survival rates in patients with CKD stage 3 to 4. **(I)** Renal survival rates in patients receiving supportive treatment. **(J)** Renal survival rates in patients who received corticosteroids alone or in combination with immunosuppressant therapy.

The subgroup analysis demonstrated a correlation between the LAR and the composite renal outcome for IgAN patients with various disease statuses, as shown in **Figure 4**. The results demonstrated that a greater LAR was still significantly associated with an increased composite renal risk in men or women (**Figures 4A, B**), in patients with proteinuria greater than 1 g/day or not (**Figures 4C, D**), in patients with or without anemia (**Figures 4E, F**), in patients with an eGFR less than 60 mL/min/1.73 m^2^ or not (**Figures 4G, H**), and in patients receiving prednisone, other immunosuppressive agents, or supportive treatment (**Figures 4I, J**).

## Discussion

In our study, the AUC of the LDH/ALB ratio (LAR) for the composite renal outcome of IgAN patients indicated that the LAR is a great index for predicting renal outcome, especially for short-term prognosis. In addition, IgAN patients with a greater LAR had more severe clinical features and more severe pathological lesions. In addition, in the multivariable Cox regression analysis, the LAR was an independent prognostic factor for the composite renal outcome of IgAN patients after adjustment for important clinicopathologic factors (such as age, gender, hypertension, renal function, anemia, renal pathological lesions, and treatment regimens). Similarly, Kaplan–Meier analysis indicated that patients with a higher LAR at baseline had a significantly greater composite renal risk. All of the results suggest that the LAR, an easily accessible and readily available metric, could be a novel independent prognostic factor for the composite renal outcome of IgAN patients.

LDH is a cytoplasmic enzyme expressed in nearly all types of cells in the body. When cells undergo injury or death caused by ischemia, starvation, dehydration, bacterial toxins, drugs, or other injuries, LDH is released into the blood, increasing the serum LDH level (15). A higher LDH level was found to be associated with greater all-cause and cardiovascular mortality in incident hemodialysis patients (16). At the same time, LDH levels are associated with concomitant kidney damage, as elevated LDH is associated with a significantly increased risk of microalbuminuria and ESRD in patients with diabetic kidney disease (17). Meanwhile, research has found that in both acute kidney injury and chronic kidney disease animal models, there is an increase in LDH levels in urine or blood samples, which is also recognized as a sign of kidney damage (18). Chronic inflammation has been demonstrated to affect the onset and progression of kidney diseases (19), especially IgAN (5). We explored biomarkers that reflect the balance between inflammatory and immune responses in IgAN (20). Inflammatory mediators are associated with renal pathologic lesions in tubulointerstitial fibrosis and renal scarring (21). At the same time, the increase in serum lactate dehydrogenase levels may indicate that a series of factors contributing to the deterioration of the patients’ conditions occur during the chronic course of the disease, such as the occurrence of acute kidney injury (22, 23) and thrombotic microangiopathy (24).

Albumin has been explored as a negative acute-phase protein in inflammatory reactions and is associated with systemic nutritional status. Thus, hypoalbuminemia or a decrease in the serum albumin concentration could be related to infection (25). There are several factors that can lead to hypoalbuminemia, including inflammation, infection, malnutrition, protein-losing disorders, oxidative stress, and liver dysfunction (26). There is no doubt that among the numerous complications of renal disease, the progressive loss of body protein mass and energy reserves is one of the most common (27). In particular, in maintaining renal perfusion and glomerular filtration, the role of albumin cannot be ignored, and additional protein loss, as well as long-term moderate and severe proteinuria, can accelerate this deterioration (28). In addition, infiltration by inflammatory cells and the presence of a large number of inflammatory factors lead to the deterioration of renal function.

In prospective studies, detection of podocyte injury-related markers and inflammatory biomarkers in the urine of IgAN patients may help clarify the relationship between LAR and molecules implicated in renal tissue, especially albumin leakage (29). Additionally, exploration of corresponding biomarkers in animal or cellular experiments is expected to further elucidate the molecular mechanisms by which LAR contributes to renal tissue injury.

The LAR has been recognized as a novel marker of inflammation in various diseases (6–8). However, to date, published studies have only explored the predictive value of the LAR for acute kidney injury caused by different conditions, such as sepsis or contrast agents, as well as its predictive value for the prognosis of death in patients with acute kidney injury (30–32), and most of the conclusions are based on data from non-Asian populations. Thus, conducting this retrospective study is of great significance, aiming to evaluate the role of LAR in predicting renal prognosis among Chinese patients with IgAN.

Alterations in lipid metabolism are detected during the progression of chronic kidney disease (CKD). Disordered lipid metabolism and other related metabolic disturbances place CKD patients at a high risk for cardiovascular disease (33). We also found that a high LAR is associated with disordered lipid metabolism and serum total cholesterol and triglyceride levels in IgAN patients.

Survival analysis revealed that patients with a high LAR had severe renal outcomes. To more accurately examine the ability of the LAR to predict the composite renal outcome, we performed different subgroup analyses. Grouped by sex, individuals with a higher LAR tended to have lower renal survival rates than those with a lower LAR among both men and women. In addition, in the subgroups of patients with different renal functions evaluated by CKD stage, patients with a higher LAR presented with greater renal risk in both CKD stages 1 to 2 and CKD stages 3 to 4. A previous clinical cohort research reported that anemic IgAN patients presented a greater risk of developing poor outcomes than nonanemic patients (34). In our subgroups of patients with or without anemia, the LAR still predicted renal risk in IgAN patients. The level of proteinuria is known to be a great index to assess the prognosis and effect of treatment strategies (35). Thus, we conducted a subgroup analysis of proteinuria, which revealed that the LAR was related to renal outcomes in patients with or without 24-h urine protein greater than 1 g. Corticosteroids have a strong anti-inflammatory effect and may influence the LAR. Therefore, we assessed the effects of corticosteroid treatment on the two groups of patients. The subgroup survival analysis suggested that the LAR could also predict disease prognosis in patients receiving supportive and corticosteroid treatment. The abovementioned results indicated that the LAR has wide applicability in predicting renal outcomes in IgAN patients.

Therefore, the LAR, a novel biomarker combining LDH and ALB that is relatively cost-effective and easily accessible, reflects both the inflammation and nutritional status of an individual and has great predictive value for renal survival.

This study had three additional limitations. First, this was a single-center retrospective study, leading to limitations in the generalizability of the results and with some inevitable bias. Our study population is predominantly from southwestern China and consists mainly of the Han ethnicity, with Tibetan and Yi ethnic groups also comprising a little proportion. Given this demographic profile, future multi-center prospective studies are warranted to validate the predictive value of LAR. Second, the mean follow-up time was relatively short (59 months), especially for patients with IgAN, which is a slowly progressing disease. Third, the longitudinal LAR data collected during patient follow-up were incomplete or contained gaps. Consequently, a more thorough longitudinal assessment of LAR could not be performed to further evaluate its predictive potential.

## Conclusion

The LAR is a significant and independent risk factor to predict disease progression and prognosis in Chinese patients with IgAN. These findings position the LAR as a practical tool for nephrologists, facilitating the rapid identification of IgAN patients with high-risk, poor-prognosis profiles. This aids in comprehensive risk stratification prior to treatment initiation. The clinical value of LAR should be validated in future multicenter studies featuring longer follow-up periods and comprehensive longitudinal data on disease progression.

## Data Availability

The raw data supporting the conclusions of this article will be made available by the authors, without undue reservation.
